# The aorta at the center

**DOI:** 10.1590/1677-5449.202300892

**Published:** 2023-09-04

**Authors:** Andre Brito-Queiroz, Grace Carvajal Mulatti, Vanessa Prado dos Santos

**Affiliations:** 1 Universidade Federal da Bahia, Hospital Ana Nery, Centro de Doenças da Aorta, Salvador, BA, Brasil.; 2 Universidade de São Paulo, Hospital das Clínicas, Departamento de Cirurgia Vascular e Endovascular, São Paulo, SP, Brasil.; 3 Universidade Federal da Bahia, Hospital Universitário Professor Edgard Santos, Departamento de Anestesiologia e Cirurgia, Salvador, BA, Brasil.

Medical progress and the huge accumulated volume of knowledge and techniques prompted the creation of medical specialties and the division of medicine into different areas, which is also the case of the surgical specialties. To a certain extent, this subdivision is even more extreme with regard to aortic surgery. Surgeons who have very different training, experience, and skills learn to manage the same vessel, but in different anatomic regions.

Routinely, cardiac surgeons are more accustomed to dealing with the aortic root and the ascending aorta, for which a sternotomy is the most common approach. In turn, vascular surgeons have greater expertise with the descending thoracic aorta and abdominal aorta, where endovascular techniques constitute the main strategy. Endovascular techniques for all segments of the aorta have developed rapidly and the state of the art in radiology, anesthesiology, intensive care, genetics, and cardiology has advanced apace.

Vascular surgery has undergone major changes over recent years, with the rapid rise of endovascular techniques. At the end of the 1980s and start of the 90s, vascular surgeons all over the world were responsible for some of the most important developments ever achieved in aortic surgery. Parodi, in Argentina, and Volodos, in Ukraine, were responsible for founding the principles of endovascular aortic surgery, constructing the first endografts by hand.^1,2^ The same principles that were initially employed in cases with favorable anatomy in the descending thoracic aorta and infrarenal aorta were then applied over the years that followed to treat practically any segment of the aorta, from the root to its bifurcation into the iliac arteries.

However, the division of the aorta into ascending and descending segments and division of the professionals who work on each segment is arbitrary and it is not rare for diseases to fail to “respect” this segmentation, involving more than one region or areas where the expertise of different specialties intersects. The best example of this is the diseases that involve the aortic arch.

The aortic arch is a transition zone between the ascending aorta and the thoracic descending aorta. The Stanford classification of aortic dissections is the most widely used worldwide and was first described in 1970. It classifies dissections involving the ascending aorta as type A and dissections restricted to the descending aorta after the origin of the left subclavian artery as type B.^3^ This left a gap in the medical literature on aortic surgery that persisted for years.^4^

This gap is not merely theoretical. Since this is a complex region that has not been entirely mastered by heart surgery, by vascular surgery, or by endovascular surgery, it is an area that was neglected for some time. In many parts of the world, vascular surgeons and heart surgeons work on opposite sides, each seeking solutions for the aortic arch alone, and also for many other situations in which diseases of the aorta involve many segments, demanding a wider and more complementary approach.

Examples of the importance of a multidisciplinary approach to the aorta are type A dissections that cause remote ischemia. In up to 40% of type A dissection cases there is also malperfusion of organs, causing an important increase in mortality among these patients. Some groups now start by treating the ischemia, dealing with the ascending aorta in a later intervention. Involving the vascular surgery team from the initial management of these cases and taking a case-by-case approach is therefore essential to provide the best possible care for these patients.^5^

In 2019 the European Society for Vascular Surgery and the European Association for Cardio-Thoracic Surgery published a consensus setting out the principal guidelines for diseases of the aorta involving the aortic arch. The first three recommendations were as follows: decision making by an aortic team is recommended; centralization of care is recommended; and treatment of elective cases is recommended to be performed in specialized centers providing open and endovascular, cardiac and vascular surgery at a single center.^6^

The importance of collaboration within a team that includes vascular surgeons, cardiac surgeons, cardiologists, radiologists, anesthetists, intensive care specialists, and, when necessary, rheumatologists, nephrologists, and geneticists would appear to be evident. The literature shows that the individual experience of the surgeon and, primarily, the institution has a positive impact on patient outcomes and many authors have demonstrated improvement of results after implementation of centers dedicated to diseases of the aorta.^7,8^

In Brazil, experience with these models is limited and implementation involves changes that are not restricted to logistic and structural issues in hospitals or operating rooms. The most important change in this setting is cultural. For many years, there was limited dialog and interaction between surgeons and clinicians, and also between cardiac and vascular surgeons. For a long period, the aortic arch was neglected by many groups, but it has now been recognized as a bridge linking the two specialties.

Without doubt, the surgeons and specialists who work in these teams and centers for treatment of the aorta will be involved in the next round of developments in surgery for this artery. Care must be based not only on anatomy, but also on collaboration, seeking solutions for diseases that are almost always complex and severe. Consolidation of these models will only be achieved through flexibility in decision-making, laying pride aside, and with dialog, modernization, and the understanding that care cannot be centralized in a specialist or a specialty, but that the aorta and the patient must be at the center of care.
