# Supplementary material for: The aorta at the center
Source: J Vasc Bras. 2023 Sep 4;22:e20230089. doi: 10.1590/1677-5449.202300892 (PMC10545235; doi:10.1590/1677-5449.202300892)
Supplement: Translation

# Translation: A aorta no centro

**DOI:** 10.1590/1677-5449.202300891

**Authors:** Andre Brito-Queiroz, Grace Carvajal Mulatti, Vanessa Prado dos Santos

**Affiliations:** 1 Universidade Federal da Bahia, Hospital Ana Nery, Centro de Doenças da Aorta, Salvador, BA, Brasil.; 2 Universidade de São Paulo, Hospital das Clínicas, Departamento de Cirurgia Vascular e Endovascular, São Paulo, SP, Brasil.; 3 Universidade Federal da Bahia, Hospital Universitário Professor Edgard Santos, Departamento de Anestesiologia e Cirurgia, Salvador, BA, Brasil.

Os progressos da medicina e o grande volume de conhecimentos e técnicas acumulados levaram à criação das especialidades médicas e de suas divisões em diferentes áreas, o que não foi diferente com especialidades cirúrgicas. Na cirurgia da aorta, essa divisão foi, de certa forma, ainda mais marcante. Cirurgiões com formações, experiências e habilidades bem distintas aprenderam a lidar com o mesmo vaso, mas em diferentes regiões anatômicas.

Efetivamente, cirurgiões cardíacos estão mais habituados a lidar com a raiz da aorta e a aorta ascendente, sendo a esternotomia a principal via de acesso; já cirurgiões vasculares têm maior domínio sobre a aorta torácica descendente e aorta abdominal, em que a via endovascular tornou-se a principal estratégia. As técnicas endovasculares evoluíram de forma rápida em todos os segmentos da aorta, assim como os conhecimentos em radiologia, anestesiologia, terapia intensiva, genética e cardiologia.

A Cirurgia Vascular tem passado por grandes mudanças nos últimos anos, com a rápida ascensão das técnicas endovasculares. Cirurgiões vasculares, ao redor do mundo, proporcionaram, no fim da década de 80 e início dos anos 90, alguns dos mais importantes avanços já realizados nas cirurgias de aorta. Parodi na Argentina e Volodos na Ucrânia foram responsáveis por forjar os princípios da cirurgia endovascular da aorta, criando, de forma artesanal, as primeiras endopróteses^1,2^ . Os mesmos princípios, que inicialmente foram utilizados em casos com anatomias favoráveis da aorta torácica descendente e aorta infrarrenal, foram utilizados nos anos subsequentes para o tratamento de praticamente qualquer segmento da aorta desde a sua raiz até a bifurcação em artérias ilíacas.

Entretanto, a divisão da aorta em ascendente e descendente, assim como a divisão dos profissionais que atuam em cada segmento, é arbitrária, e não raramente as doenças não “respeitam” essas segmentações e acometem mais que um território ou até mesmo territórios de intersecção entre as especialidades. O maior exemplo disso são as doenças que acometem o arco aórtico.

O arco da aorta é uma zona de transição entre a aorta ascendente e a aorta torácica descendente. A classificação de Stanford para dissecções da aorta é a mais utilizada em todo o mundo, tendo sido descrita inicialmente em 1970. Ela classifica como tipo A as dissecções que envolvem a aorta ascendente e tipo B as dissecções que se restringem à aorta descendente após a origem da artéria subclávia esquerda^3^ . Ficou, assim, estabelecida uma lacuna na literatura médica, que perdurou por anos na cirurgia da aorta^4^ .

Essa lacuna não é meramente teórica. Por se tratar de um território complexo, não completamente dominado pela cirurgia cardíaca, nem sequer pela cirurgia vascular ou endovascular, esta área foi por muito tempo negligenciada. Em diversos locais do mundo, cirurgiões vasculares e cirurgiões cardíacos trabalharam em lados opostos, buscando sozinhos as soluções para o arco aórtico, assim como para várias outras situações em que as doenças da aorta acometem diversos segmentos e necessitam de uma abordagem mais ampla e complementar.

Exemplos da importância da abordagem multidisciplinar na aorta são as dissecções do tipo A que resultam em quadros isquêmicos a distância. Em até 40% dos casos de dissecção tipo A, há também má perfusão de órgãos, que resulta em importante elevação da mortalidade nesses pacientes. Alguns grupos têm realizado inicialmente o tratamento do quadro isquêmico, com a abordagem da aorta ascendente em um segundo momento. O envolvimento da cirurgia vascular desde o início desses atendimentos e uma abordagem individualizada dos pacientes é, portanto, fundamental para uma melhor condução desses casos^5^ .

Em 2019 a Sociedade Europeia de Cirurgia Vascular e a Associação Europeia de Cirurgia Cardiotorácica publicaram um consenso com as principais diretrizes para as doenças da aorta envolvendo o arco aórtico. As três primeiras recomendações foram as seguintes: a tomada de decisão deve ser realizada por um time de aorta; a centralização do cuidado é recomendada; e o tratamento dos casos eletivos deve ser realizado em serviços especializados, que ofereçam cirurgia aberta e endovascular, cardíaca e vascular em um único centro^6^ .

A importância da colaboração de um time que envolva cirurgiões vasculares, cirurgiões cardíacos, cardiologistas, radiologistas, anestesistas, intensivistas e, quando necessário, reumatologistas, nefrologistas e geneticistas parece ficar evidente. A literatura mostra que a experiência individual do cirurgião e principalmente da instituição tem impacto positivo no desfecho dos pacientes, e diversos autores têm demonstrado melhoria nos resultados após a implementação de centros dedicados às doenças da aorta^7,8^ .

No Brasil, a experiência com esses modelos é pequena, e essa implementação envolve mudanças não somente logísticas e estruturais nos hospitais ou salas de cirurgia. A principal mudança nesse cenário é cultural. Por muitos anos, o diálogo e a interação entre cirurgiões e clínicos, assim como entre cirurgiões cardíacos e vasculares, foram limitados. O arco da aorta, que por um longo período foi negligenciado por muitos grupos, representa hoje uma ponte que liga as duas especialidades.

Certamente, os cirurgiões e demais especialistas que se associarem a grupos e centros de cuidado da aorta vivenciarão os próximos avanços da cirurgia dessa artéria. Esse cuidado deve ser baseado não somente na anatomia, mas na colaboração e busca de soluções para doenças quase sempre complexas e graves. A concretização destes modelos só será possível com flexibilidade nas decisões, despimento de vaidades, diálogo, atualização e entendimento de que o cuidado não deve ser centralizado em um médico ou em uma especialidade, mas que a aorta e o paciente precisam estar no centro do cuidado.
